# Burden and Challenges of Managing Hypertension in People Living with Human Immunodeficiency Virus (HIV) Infection in Sub-Saharan Africa: A Mixed Systematic Review and Meta-Analysis

**DOI:** 10.3389/ijph.2025.1608521

**Published:** 2025-12-17

**Authors:** Francis Duhamel Nang Nang, Liliane Mfeukeu Kuate, Anastase Dzudie, Fabrice Djouma Nimbot, Paul Junior Chebo, Luc Baudoin Fankoua Tchaptcha, André Pascal Kengne, Jean Pierre Junior Tchitetchoun, François Anicet Onana Akoa, Rita Marie Ifoue, Lawson Ngwagwe Mbolueh, Charles Kouanfack, Simeon Pierre Choukem

**Affiliations:** 1 Department of Public Health, Faculty of Medicine and Pharmaceutical Sciences, University of Dschang, Dschang, Cameroon; 2 CARE International in Cameroon, Yaoundé, Cameroon; 3 Cardiology Unit, Yaoundé Central Hospital, Yaoundé, Cameroon; 4 Faculty of Medicine and Biomedical Sciences, University of Yaoundé I, Yaoundé, Cameroon; 5 Department of Medicine, University of Cape Town, Cape Town, South Africa; 7 Clinical Research Education, Networking and Consultancy (CRENC), Yaoundé, Cameroon; 7 Faculty of Medicine and Pharmaceutical Sciences, University of Douala, Douala, Cameroon; 8 Douala General Hospital, Douala, Cameroon; 9 Faculty of Health Sciences, University of Buea, Buea, Cameroon; 10 African Population and Health Research Center (APHRC), Nairobi, Kenya; 11 International Center for Multidisciplinary Research, University of Lisala, Lisala, Democratic Republic of Congo; 12 Department of Public Health, Catholic University of Central Africa, Yaoundé, Cameroon; 13 SOLTHIS Sierra Leone, Freetown, Sierra Leone; 14 Yaoundé Central Hospital, Day Hospital, Yaoundé, Cameroon; 15 Center for Research on Emerging and Re-Emerging Diseases (CREMER), Yaoundé, Cameroon; 16 Research Network on Health and Human Development (2HD), Douala, Cameroon; 17 Harris Manchester College, University of Oxford, Oxford, United Kingdom

**Keywords:** hypertension, HIV, people living with HIV, sub-Saharan Africa, systematic review, meta-analysis, care cascade, integrated care

## Abstract

**Objectives:**

Poor hypertension prevention among people living with HIV (PLHIV) in sub-Saharan Africa may undermine progress in HIV prevention achieved within this population. This review synthesizes data on the prevalence, diagnosis, treatment, control, and challenges related to hypertension prevention among PLHIV in SSA.

**Methods:**

A mixed-method systematic review with meta-analysis was conducted from January to December 2024. Data analysis was performed using random-effects modeling stratified by age and sex, along with thematic analysis. The JBI critical appraisal tool was used to assess study quality.

**Results:**

A total of 31 studies, including 32286 PLHIV (±37 years old), were included. Hypertension affected 25% [95% CI, 21%–28%] of PLHIV, yet only 34% [95% CI, 11%–64%] of cases received treatment, and 19% [95% CI, 3%–34%] had controlled blood pressure, despite BP measurement in 95% [95% CI, 89%–100%] of them.

**Conclusion:**

Hypertension prevention challenges stem from patient-related factors, healthcare providers, and health system gaps. Despite the high prevalence of hypertension among PLHIV, treatment initiation and blood pressure control rates remain low. Strengthening the integration of HIV and hypertension care services is urgently needed.

## Introduction

The widespread use of antiretroviral therapy (ART) has led to viral suppression in 92% of people living with HIV in sub-Saharan Africa [1], resulting in a 68% reduction in AIDS-related deaths globally and 64% in sub-Saharan Africa. These improvements have narrowed the life expectancy gap between people living with HIV (PLHIV) and those without HIV [2–4]. However, the increased life expectancy of PLHIV has led to a rise in non-communicable diseases, particularly cardiovascular diseases (CVDs) [4–6], with hypertension being a major concern [7–9]. HIV-positive adults on ART are more likely to have hypertension than those without HIV [10], with 35% of ART users globally affected, compared to 30% of HIV-negative adults [4]. In sub-Saharan Africa, the prevalence of hypertension among PLHIV is well-documented, with studies showing 25% in Cameroon and 24.3% in Uganda [4, 8, 11–13]. Despite this, treatment initiation and control rates remain low [14]. A preliminary review of existing research revealed no mixed-method systematic review on this topic. However, a systematic review with meta-analysis addressing the issues of prevalence, awareness, treatment, and control of hypertension, and the availability of hypertension prevention services for people living with HIV in sub-Saharan Africa (SSA) was found [15]. The authors of this review reported the high prevalence of hypertension in a relatively young population of PLHIV, with suboptimal screening, treatment, and control of hypertension. This study will evaluate the prevalence, diagnosis, treatment, control, and challenges of hypertension prevention among PLHIV in sub-Saharan Africa.

What is the burden of hypertension, and the challenges encountered in the prevention cascade among people living with HIV (PLHIV) in sub-Saharan Africa?

- What is the prevalence of hypertension among PLHIV in sub-Saharan Africa?
- What are the proportions of treatment initiation and blood pressure control among PLHIV with hypertension in sub-Saharan Africa?
- What are the challenges in the hypertension treatment cascade among adults enrolled in HIV care in sub-Saharan Africa?

## Methods

### Protocol and Registration

This review follows the Joanna Briggs Institute (JBI) methodology for mixed-methods systematic reviews (MMSR) [16]. This protocol has been registered in PROSPERO (CRD42023418363). Details on study evaluation, statistical analysis, and definitions are outlined below.

Study Eligibility CriteriaAs part of this mixed-methods systematic review (MMSR), only studies specifically addressing the issue of hypertension among people living with HIV (PLHIV) were included. Inclusion criteria were defined separately for the quantitative and qualitative components.

### Quantitative Component

Eligible studies included those conducted in sub-Saharan Africa involving PLHIV aged 18 years and above, with blood pressure measured at least twice for hypertension screening or monitoring. Studies assessing the prevalence, diagnosis, treatment, or control of hypertension regardless of participants’ ethnicity, socioeconomic status, or education level were included. All types of quantitative study designs and related data were eligible.

### Qualitative Component

Qualitative studies were included if they explored barriers and facilitators to hypertension prevention, diagnosis, treatment, or management among PLHIV or healthcare providers in SSA. All methods of data collection (e.g., interviews, focus groups, observations) and all analytical approaches (e.g., thematic analysis, grounded theory, content analysis) were eligible, provided the study offered insights into hypertension care among PLHIV.

Information Sources and Search Strategy The search aimed to identify both published and unpublished studies using a three-step strategy. It began with a limited search of PubMed-MEDLINE, followed by an analysis of titles, abstracts, and indexing terms. Keywords such as “challenges,” “prevalence,” “treatment,” “control,” “hypertension,” and “HIV,” along with an African filter by Siegfried, were used to identify studies conducted in sub-Saharan Africa (SSA). Database subject headings (MeSH in PubMed/MEDLINE, CINAHL, and Google Scholar) were combined with the names of African countries in English and relevant languages. The search was limited to original research articles published in Africa between January 1, 2000, and May 30, 2024, in English and Frenc.

### Study Selection

The citations were gathered in Rayyan [17], and duplicates were removed. Two independent reviewers (FDNN and RME) reviewed the selected articles based on predefined eligibility criteria, including title, abstract, and full text quality. Discrepancies were resolved through discussion, with a third reviewer (CPJ) involved in the final selection. The methodological quality of the studies was evaluated using the MMAT tool, French version 2018 [18]. This evaluation ensured the scientific validity of the studies and their adherence to methodological standards. Each evaluation question was answered with “yes,” “no,” or “I do not know,” applied to the selected full-text articles to determine eligibility and assess study quality. Responses of “no” or “I do not know” indicated that the study may not be scientific.

### Data Extraction

Data were extracted by two independent reviewers and recorded in a standardized form using the Comprehensive Meta-Analysis data extraction tool [19]. Qualitative data were extracted using the JBI SUMARI tool.

#### Quantitative Data Was Extracted

For quantitative studies (and the quantitative components of mixed-methods studies), data were systematically extracted using the Comprehensive Meta-Analysis (CMA) software and organized in an Excel 2019 database to facilitate management and analysis. Two independent reviewers carried out the initial data extraction, followed by verification by a second reviewer to ensure consistency and data quality.

The extracted data included:

- Article details: study authors, year of publication, year of data collection, and study setting (e.g., urban or rural, primary or specialized care).
- Study characteristics: country of implementation, study design (cross-sectional, case-control, cohort, etc.), target population (e.g., adults living with HIV, general population), sample size, and sampling method (probability or non-probability).
- Definition of hypertension (HTN): criteria for defining HTN were standardized according to the European Society of Cardiology (ESC) and the European Society of Hypertension (ESH) guidelines [19], namely systolic blood pressure (SBP) ≥ 140 mmHg and/or diastolic blood pressure (DBP) ≥90 mmHg and/or current use of antihypertensive medication. These uniform definitions allowed comparability across studies [19].
- Indicators of hypertension care: this included data on the prevalence of HTN among people living with HIV (PLHIV), the treatment rate (proportion of individuals diagnosed with HTN and receiving antihypertensive therapy), and the control rate (proportion of treated individuals achieving BP control as defined by ESC/ESH thresholds).

This standardized approach to quantitative data extraction was designed to allow for inter-study comparison and potential meta-analysis. It aligns with PRISMA guidelines and follows the recommendations of the Cochrane Handbook for Systematic Reviews of Interventions for rigorous and transparent data collection in systematic reviews [20].

#### Qualitative Data Were Extracted and Analysis

Qualitative data were extracted using the JBI System for the Unified Management, Assessment and Review of Information (JBI SUMARI) software, which provides a standardized framework for the synthesis of qualitative evidence [21]. For qualitative studies (and the qualitative components of mixed-methods studies), the extracted information included detailed data on the study population, contextual and cultural setting, geographic location, study methods, and the phenomenon of interest, namely barriers and facilitators to hypertension care among PLHIV in SSA. Thematic findings relevant to healthcare access, patient-provider interactions, knowledge and beliefs about hypertension, structural health system barriers, and stigma were recorded. Each finding was extracted alongside its supporting illustrations (participant quotes or field observations), and a level of credibility was assigned based on JBI criteria—categorized as “unequivocal,” “credible,” or “unsupported” [22].

This approach to qualitative data extraction aligns with best practices in evidence synthesis for complex public health topics, allowing for nuanced interpretation of individual and contextual factors influencing hypertension care in vulnerable populations [22, 23]. The use of JBI SUMARI further ensures methodological rigor in the extraction and appraisal of qualitative evidence, enhancing the validity and reproducibility of the review findings [24, 25].

The qualitative studies included in this review primarily employed inductive and interpretive methods to explore the experiences and perceptions of PLHIV and healthcare providers regarding hypertension care in sub-Saharan Africa. Data collection methods included semi-structured individual interviews [23], focus group discussions to capture social norms and shared perceptions [26], and, less commonly, direct or participant observations to contextualize clinical practices and patient-provider interactions.

The main themes explored included knowledge and perception of hypertension, perceived stigma in integrated HIV-HTN services, structural and organizational barriers (e.g., costs, distance, staff shortages, drug stockouts), provider attitudes, and patient preferences regarding service delivery.

All types of qualitative analysis approaches were eligible, including thematic analysis [25], content analysis, and more interpretive frameworks such as phenomenology and grounded theory [23]. Extracted findings were categorized by credibility level (unequivocal, credible, unsupported) in accordance with JBI standards [21]. This process helped capture the multifaceted individual, social, and systemic factors shaping hypertension care among PLHIV and identified leverage points for improving integrated care delivery.

Data Synthesis Methods Quantitative and qualitative data were analyzed separately according to the JBI methodology for mixed-method systematic reviews using JBI SUMARI [16]. Each study was critically appraised using the Mixed Methods Appraisal Tool (MMAT), with each question answered as “Yes,” “No,” or “I do not know.” A response of “No” or “I do not know” indicated that the study might not meet scientific standards, or that there was insufficient information to draw definitive conclusions. In these cases, further clarification was sought through additional documents or by contacting the authors directly.

### Meta-Analysis

We conducted a meta-analysis of the data collected in the systematic review, focusing primarily on the meta-analysis of proportions. Four cases were considered: (1) the global estimate of the prevalence of participants screened for hypertension in the selected studies; (2) the estimate of the proportion of people living with HIV diagnosed with hypertension; (3) the estimate of PLHIV with hypertension receiving care for hypertension; and (4) the proportion of PLHIV with controlled hypertension. The random-effects method was used to estimate heterogeneity between studies, measured using I^2^ statistics [27] and the tau-squared coefficient. Ninety-five percent confidence intervals were obtained to indicate precision around the estimated percentage after applying transformations to individual proportions. Due to the typically observed asymmetrical distribution for proportions, we used the Freeman-Tukey method [28], based on a double arcsine transformation, to adjust the data to resemble a normal distribution. Furthermore, an influence analysis was performed using tests to identify studies with extreme differences. In cases of highly influential studies, these were excluded, and the overall effect was recalculated based on the remaining studies. The Der Simonian and Laird method, 1986, adapted for proportions, was used for the meta-analysis [29]. When heterogeneity persisted, we conducted a meta-regression using study type as the main variable. A challenge at this stage was the small number of studies, typically fewer than nine, depending on the indicators studied. Several authors have recommended conducting a meta-regression with at least ten studies while controlling for study-related variables [30–33]. Finally, forest plots were presented to visualize the results. The related variables were study type, publication year, age group, and study size. However, due to the small sample sizes of the studies and for decision-making purposes, we selected the age group. Asymmetry between studies was also tested using a normal distribution. In the context of proportion meta-analyses, publication bias is considered less informative than in randomized trials [34]. However, we tested asymmetry between studies using the normal distribution law.

A random-effects model was used to pool study-specific estimates of hypertension prevalence. Study heterogeneity was assessed using the I^2^ statistic, with values of 0%–25%, 26%–50%, and >50% indicating low, moderate, and high heterogeneity, respectively. Heterogeneity sources were explored by comparing hypertension prevalence between subgroups based on setting (e.g., gender, age group). The pooled prevalence was compared between the random-effects model and the fixed-effects model. Publication bias was assessed through funnel plots, and an influence analysis was conducted to identify any studies with extreme values that could significantly affect the overall effect [35]. In cases where highly influential studies were identified, these were excluded, and the analysis was recalculated with the remaining studies. Meta-regression was used to explore sources of heterogeneity when needed, focusing primarily on study type.

### Meta-Aggregation

Qualitative findings (including the qualitative components of mixed-methods studies) were synthesized using the meta-aggregation approach of the JBI, through the JBI SUMARI (System for the Unified Management, Assessment and Review of Information) software. This method provided a rigorous and standardized framework for aggregating qualitative evidence, facilitating its interpretation within the context of evidence-based practice [23].

This mixed-methods systematic review followed the JBI meta-aggregation approach, as outlined in the JBI Manual for Evidence Synthesis. Qualitative findings, including the qualitative arms of mixed-methods studies, were synthesized using a three-step process aimed at preserving the original meaning of participants’ experiences and minimizing re-interpretation.

Extraction of findings: Qualitative findings relevant to the review question were extracted verbatim from the results sections of each included study. These were accompanied by illustrative supporting quotes and assigned a level of credibility (unequivocal, credible, or unsupported) in accordance with JBI guidance.

Development of categories: The extracted findings were grouped into descriptive categories based on similarity in meaning. Categories were developed inductively by two independent reviewers through thematic clustering and refined through discussion and consensus.

Synthesis of findings: The descriptive categories were then synthesized into overarching analytical findings, reflecting a higher level of abstraction and generalizability. The synthesis aimed to generate actionable insights for public health practice and policy, particularly within HIV service delivery in sub-Saharan Africa.

Where aggregation was not feasible due to heterogeneity in context or phenomenon of interest, findings were summarized narratively. All steps of the synthesis were conducted using a transparent, auditable process within a JBI-aligned Excel matrix. No software was used beyond Microsoft Excel. Disagreements between reviewers were resolved through discussion, or by involving a third reviewer.

The synthesis ultimately generated five integrated findings, structured across three levels of analysis: patient-level, provider-level, and health system-level barriers to hypertension prevention among people living with HIV (PLHIV) in sub-Saharan Africa [36].

## Results

### Study Inclusion

**FIGURE 1:**
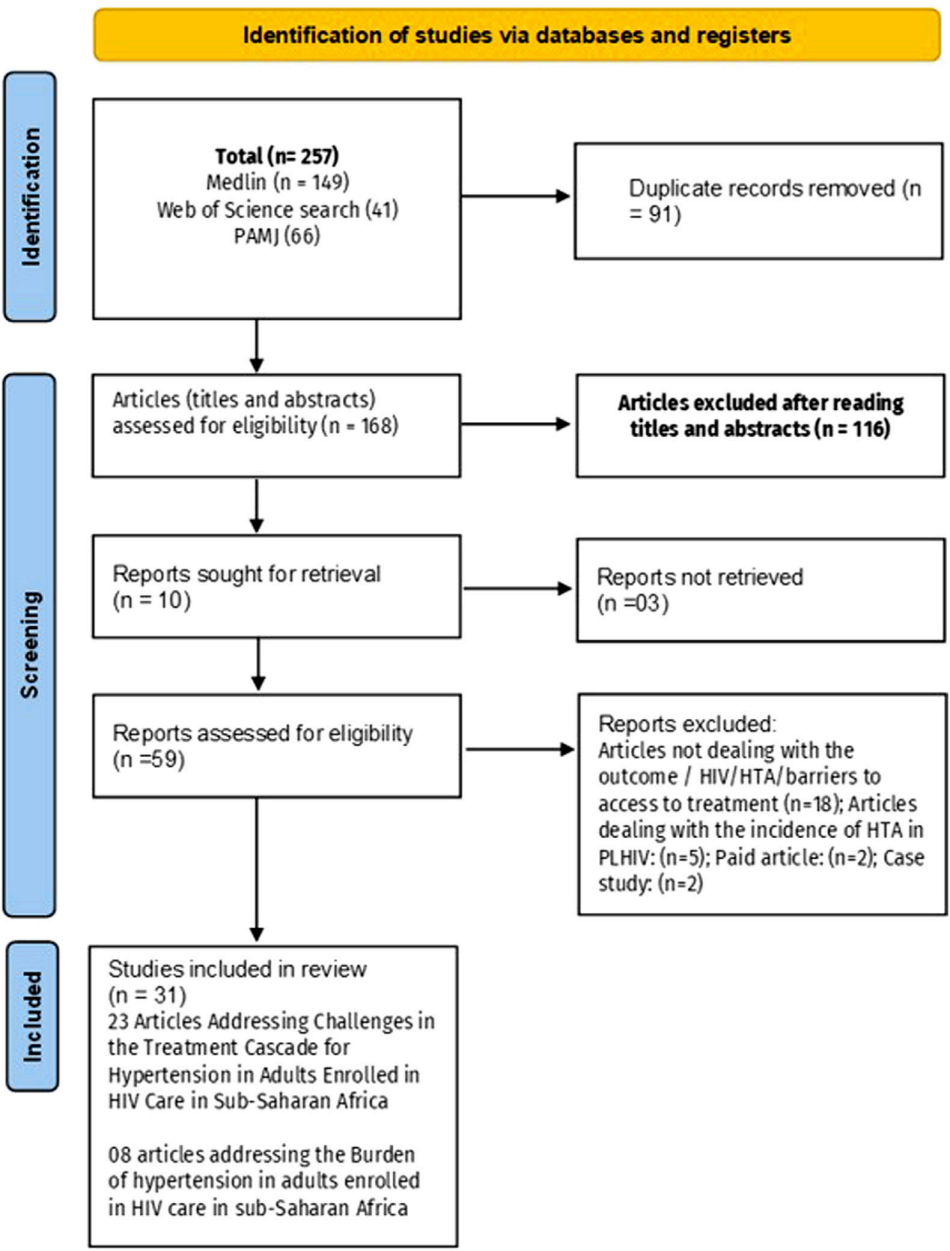
Flowchart summarizing the process for including eligible studies (Mixed systematic review and meta-analysis, sub-Saharan Africa, 2017–2024).

After duplicates were removed from the 257 identified records, 168 titles and abstracts were screened, and 55 full-text articles were assessed for eligibility. Ultimately, 31 studies were included: 23 for the quantitative synthesis and 8 for the qualitative synthesis (Figure 1). The meta-analysis focused on four main outcomes: the prevalence of hypertension among PLHIV, the proportion with access to blood pressure measurement, the proportion of hypertensive PLHIV receiving treatment, and the proportion achieving blood pressure control.

For the qualitative synthesis, findings were extracted and analyzed using the JBI meta-aggregation approach. Themes related to barriers and challenges in hypertension care among PLHIV in Sub-Saharan Africa were categorized and synthesized. Main themes included service availability, access barriers, healthcare provider attitudes, patient preferences, and systemic limitations. These findings provided a comprehensive understanding of the factors influencing hypertension care delivery.

### General Characteristics of the Included Studies

The 31 included studies (quantitative and qualitative) were published between 2017 and 2024 across 15 Sub-Saharan African countries (Table 1). Sample sizes ranged from 13 to 77,696 participants, with the proportion of men varying between 23% and 85%. The mean age of participants ranged from 35 to 50 years, with an overall average of 41.6 years. The weighted proportion of women was 72%. A total of 23 studies assessed the burden of hypertension among people living with HIV (PLHIV), while 13 studies addressed challenges related to hypertension prevention in this population. The methodological quality scores, based on the Mixed Methods Appraisal Tool (MMAT) [19, 24, 27], ranged from 5 [39] to 7 [24]. Fifteen studies reported the proportion of PLHIV who had been screened for hypertension, 10 studies presented data on hypertension treatment among PLHIV [11, 14, 38, 39, 42, 49, 50, 62–64], and 5 studies provided data on blood pressure control [14, 38, 49, 63].

**TABLE 1:** Baseline characteristics of included studies (Mixed systematic review and meta-analysis, sub-Saharan Africa, 2017–2024).

Authors Year and references	Type of study	Country	N
Alemu Gebrie [37]	Cross-sectional	Ethiopia	407
Dzudie et al. [11]	Cross-sectional	Cameroon	9988
Hoffman et al. [38]	Prospective cohort	Malawian	671
Manavalan et al. [39]	Cross-sectional	Tanzania	806
Fiseha et al. (2019) [40]	Cross-sectional	Ethiopia	408
Sewale et al. [41]	Cross-sectional	Ethiopia	412
Sakita et al. [42]	Prospective_ cohort	Tanzania	500
Robert Musekwa et al. [43]	Cross-sectional	Zambia	348
Lubega et al. (2021) [44]	Retrospective analysis	Uganda	2026
Byonanebye et al. [45]	Prospective cohort	Uganda	1000
Grace_Wambura_Mbuthia et al. [46]	Cross-sectional	Kenya	939
Chiwandire et al. [47]	Cross-sectional	South_Africa	2327
Sarfo et al. [48]	Cross-sectional	Ghana	451
William Kudzi et al. [37]	Cross-sectional	Ghana	308
Harimenshi et al. [49]	Cross-sectional	Burundi	1250
Trifirò et al. [50]	Retrospective analysis	Tanzania	242
Lubega et al. [44]	Retrospective cohort	Uganda	2026
Muddu et al. [51]	Cross-sectional	Uganda	1649
Idongesit et al. [44]	Cross-sectional	Nigeria	417
Uwanyirigira et al. [52]	Longitudinal	Rwanda	406
Lartey [53]	Cross-sectional	Ghana	5030
Denu [54]	Cross-sectional	Ghana	362
Mutemwa [55]	Qualitative	South_Africa	827
Tiward et al. [56]	Qualitative	Tanzania	16
Gooden et al. [57]	Prospective cohort	Tanzania	26
Hing et al. [38]	Cross-sectional	Malawians	75
Manavalan et al. [39]	Qualitative	Malawians	158
Godongwana et al. [58]	Cross-sectional	Tanzania	555
Manavalan et al. [59]	Qualitative	Tanzania	13
Kagaruki et al. [60]	Qualitative	Tanzania	754
Godongwana et al. [58]	Cross-sectional	South Africa	24
Manavalan et al. [61]	Cross-sectional	Tanzania	15

### Meta-Analyses

**FIGURE 2:**
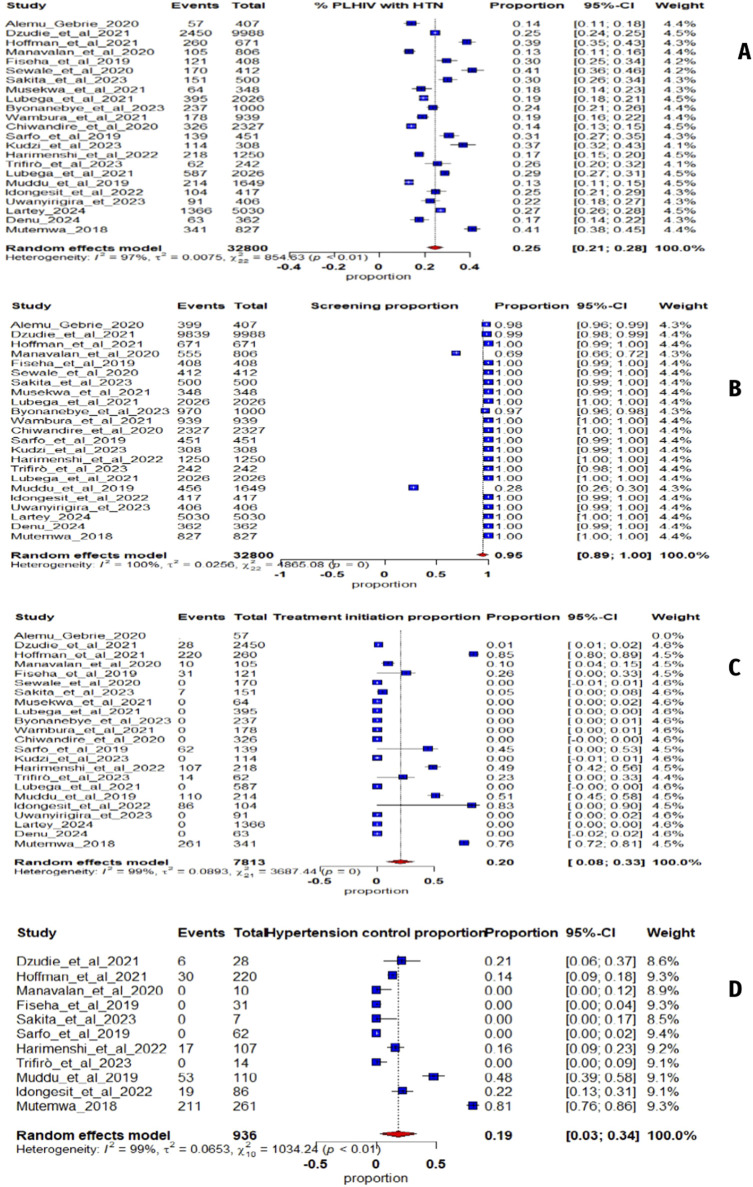
**(A)** Forest plot illustrating the prevalence of hypertension in sub-Saharan Africa from 23 studies; **(B)** Screening hypertension proportion whit People Living with Human Immunodeficiency Virus; **(C)** Proportion of People Living with Human Immunodeficiency Virus with hypertension receiving antihypertensive treatment; **(D)** Proportion of People Living with Human Immunodeficiency Virus on antihypertensive treatment (Mixed systematic review and meta-analysis, sub-Saharan Africa, 2017–2024).

**FIGURE 3:**
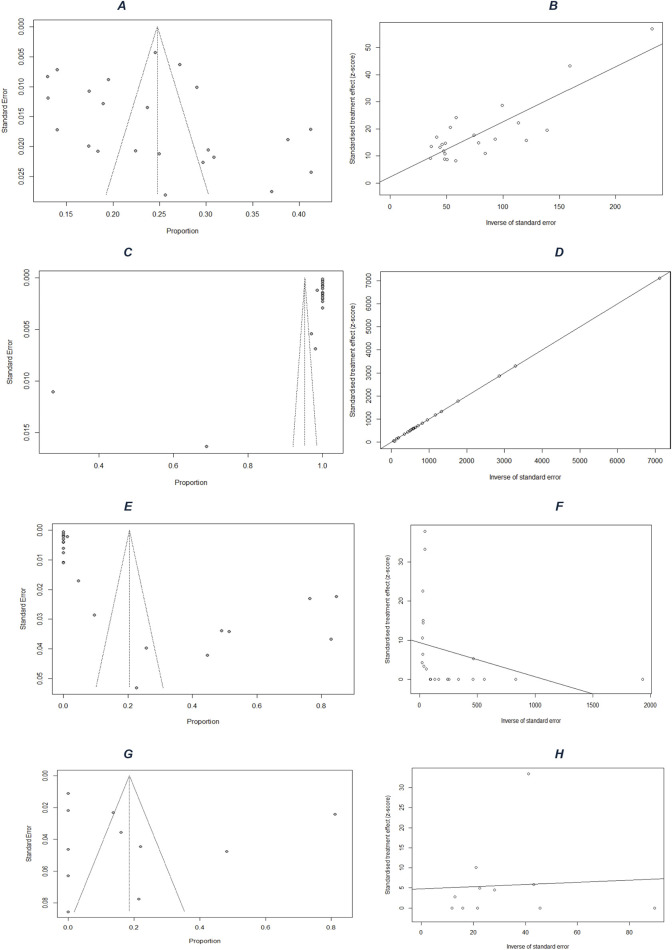
Publication-bias assessment: funnel plots for **(A)** prevalence, **(C)** screening, **(E)** treatment, and **(G)** control; and Egger’s regression plots for **(B)** prevalence, **(D)** screening, **(F)** treatment, and **(H)** control (Mixed systematic review and meta-analysis, sub-Saharan Africa, 2017–2024).

The studies show that among 32,800 PLHIV (72% women), the average hypertension prevalence in sub-Saharan Africa is 25% (95% CI: 21%–28%, I^2^ = 97%) (Figure 2A). The funnel plot (Figure 3A) confirms heterogeneity, while Egger’s plot (Figure 3B) shows a regression line near zero, indicating no publication bias (subgroup p-values: 0.75 for age, 0.13 for gender) (Supplementary Figures S1, S2).

For hypertension screening, an estimated 95% (95% CI: 89%–100%, I^2^ = 100%) (Figure 2B) of PLHIV were screened. The funnel plot (Figure 3C) confirms heterogeneity, and Egger’s plot (Figure 3D) shows no publication bias (p-values: 0.39 for age, 0.55 for gender) (Supplementary Figures S3, S4).

Regarding treatment, 20% (95% CI: 8%–33%, I^2^ = 99%) (Figure 2C) of hypertensive PLHIV initiated and remained compliant with antihypertensive treatment. The funnel plot (Figure 3E) supports heterogeneity, while Egger’s plot (Figure 3F) indicates potential publication bias. Subgroup analysis yielded a significant p-value for age (0.04) but not for gender (0.52) (Supplementary Figures S5, S6).

Finally, among six studies on blood pressure control, 19% (95% CI: 3%–34%, I^2^ = 99%) (Figure 2D) of treated hypertensive PLHIV achieved control. The funnel plot (Figure 3G) confirms heterogeneity, and Egger’s plot (Figure 3H) is horizontal, indicating no publication bias. Subgroup p-values were 0.09 for age and 0.02 for gender (Supplementary Figures S7, S8).

### Meta-Aggregation

- A meta-aggregation of qualitative findings from 08 studies conducted between 2017 and 2024 in SSA revealed a multidimensional set of barriers affecting hypertension prevention among PLHIV. The challenges were categorized across three interrelated levels: individual (patient), provider, and health system. Patient-Level Barriers
  1. Limited knowledge about hypertension: Many PLHIV lacked awareness regarding hypertension symptoms, complications, risk factors, and prevention strategies. Confusion between HIV and hypertension was frequent, and hypertension was sometimes perceived as a curse or a disease only treatable by traditional healers.
  2. Misperceptions and cultural beliefs: The fear of HIV-related stigma led some PLHIV to adopt high-fat diets to maintain or gain weight, perceiving thinness as a signal of HIV infection. Herbal medicine was commonly used as a cheaper, more accessible alternative.
  3. Psychological and behavioral barriers: Depression, stress, and HIV-related stigma discouraged engagement in preventive care. Patients reported forgetfulness, intentional treatment interruption when they felt better, and fear or experience of side effects.
  4. Therapeutic burden: The polypharmacy associated with HIV care made it difficult to integrate antihypertensive treatment, which was often seen as an added burden.
  5. Financial constraints: Patients frequently reported being unable to afford antihypertensive medication, clinical consultations, laboratory tests, or transport costs to health facilities.
- Provider-Level Barriers
  1. Insufficient training in hypertension care: Providers often lacked awareness of the specific burden of hypertension among PLHIV and had not received adequate training or continuing education.
  2. Inadequate patient counseling and communication: Communication between providers and patients was often unclear or ineffective. Counseling on hypertension was frequently absent or poorly adapted to patients’ understanding.
  3. Suboptimal prescribing practices: Ineffective drug regimens, lack of individualized treatment plans, and poor follow-up on medication adherence were reported in several settings.
- Health System-Level Barriers
  1. Fragmentation of HIV and hypertension services: Hypertension screening and care were often offered in separate services from HIV care, generating additional costs and logistical barriers for patients.
  2. Shortages of resources: Antihypertensive medications were frequently unavailable. Blood pressure monitoring equipment was lacking in many HIV care centers.
  3. Limited accessibility: Costs of care were often not covered by public systems. Long distances to health facilities, long waiting times, and insufficient social support were also commonly reported.
  4. Lack of standardized protocols: There were no formal guidelines for hypertension screening, referral, and follow-up in most HIV care settings.
  5. Poor health information systems: Weak data collection and patient tracking systems limited the ability to monitor blood pressure and ensure long-term follow-up.

Hypertension prevention among PLHIV in sub-Saharan Africa is hindered by a complex interplay of structural, professional, and individual-level barriers. These challenges compromise access, adherence, continuity, and the overall effectiveness of preventive care. Strengthening the integration of HIV and non-communicable disease services, promoting health education, and enhancing provider training are crucial for improving hypertension prevention and control among PLHIV in this setting. Nevertheless, these challenges have been classified in (Table 2) below by included article, to ensure reproducibility in the event of a possible future analysis.

**TABLE 2:** Classification of patient-level, provider-level and health system-level barriers to hypertension prevention among people living with HIV, by included study consolidated framework for future reproducibility and comparative analysis (Mixed systematic review and meta-analysis, sub-Saharan Africa, 2017–2024).

DOI	Authors	Challenges in the hypertension treatment cascade
https://doi.org/10.1186/s12913-024-10688-8	Ottaru et al. [56]	- Limited availability of hypertension screening services at HIV treatment centers - Lack of antihypertensive medications at HIV care and treatment centers (CTCs) - Perceived complexity of managing hypertension alongside HIV care - Lack of appropriate patient education to address the care needs of HIV-related comorbidities - High cost of hypertension medications makes them unaffordable - High consultation costs at hypertension clinics - Fear of side effects from hypertension medications - Perception that hypertension medications are ineffective - High transportation costs to healthcare facilities - Use of herbal remedies, perceived as a cheaper alternative for controlling hypertension - Self-monitoring of symptoms at home, eliminating the need to visit health facilities - Irregular use of medications
https://doi.org/10.1186/s12889-023-17069-6	Gooden et al. [57]	- Organizational and health system factors: (Fragmented HIV and NCD services; No protocols for NCD screening; lack of access to diagnostic equipment; lack of continuity of care for NCDs.) - Individual factors: (Health professionals’ knowledge of NCDs; PLHIV’s knowledge of NCDs; self-monitoring of NCDs) - Syndemic factors: (Poverty among PLHIV; HIV-related stigma; mental health issues among PLHIV)
10.1093/Heapol/czz112. PMID: 31723966; PMCID: PMC7967790	Hing et al. [61]	- Obstacles related to the financial aspects of hypertension medications - Shame of having to ask loved ones for financial support - Transportation costs to access hypertension treatment clinics - The high cost of hypertension exams - Lack of a national policy for managing non-communicable diseases (NCDs) - The burden of taking multiple medications daily - Side effects of medications - Changes in lifestyle, including the adoption of a new diet.
https://doi.org/10.5334/gh.1081	Hoffman et al. [38]	- Barriers to taking antihypertensive medications (not having enough money to buy antihypertensive medications (58.1%, n = 43); difficulty remembering to take medications every day (39.2%, n = 29); Lack of funds for transportation to the clinic for refills (36.5%, n = 27); Feeling that medications were no longer needed due to good health (29.7%, n = 22) - Factors associated with uncontrolled blood pressure (self-reported non-adherence to antihypertensive medications)
https://doi.org/10.1111/jch.13929	Manavalan et al. [39]	- Lack of awareness of HTN by providers;
https://doi.org/10.1371/journal.pone.0243059	Manavalan et al. [59]	- Limited knowledge of hypertension, including what it is, its causes, progression, symptoms, complications, and treatment - Participants believe that controlling stress is a better way to control hypertension - Perceived overlap and comparisons between hypertension and HIV - Delayed linkage to care after their initial hypertension diagnosis or were not linked to care at all - Difficulties with communication and counseling with providers - Reluctance to take antihypertensive medications - Lack of integration for hypertension and HIV care - Additional structural barriers to hypertension care
https://doi.org/10.1186/s12889-018-5639-7	Kagaruki et al. [60]	- Knowledge of HBP by PLHIV: - Low knowledge of HBP risk factors, prevention strategies and associated complications - Low knowledge of hygiene and dietary measures for the prevention of HBP. - Low knowledge of dietary measures to prevent HBP. - Patients claim that ARVs increase the occurrence of HBP. - Lack of health education provided in HIV clinics - Perception of PLHIV of HBP: - PLHIV thought they had to be fat because if you become thin, you will suffer from HIV-related stigma - PLHIV also thinks that eating foods rich in fat and carbohydrates to increase or maintain their weight - An incorrect perception of HBP. People living with HIV perceive HBP as a disease that is treated by traditional practitioners and that it would be a curse for their mistakes
https://doi.org/10.1186/s12913-021-06670-3	Goodgame et al. [58]	- Underfunded facilities (lack of staff; slow service delivery; drugs not delivered on time; lack of information provided to patients on prevention and management of chronic diseases; lack of HIV and chronic disease support programs; lack of training and orientation on chronic care delivery; No training provided to health professionals on HIV and chronic disease treatment and care; health professionals are not available for training on integrated chronic care); - Non-disclosure (non-disclosure of HIV status; stigma; failure to take treatment) - Polypharmacy (difficulty taking more than one treatment; pill load) - Poor knowledge of treatments (names of treatments taken; understanding chronic medications) - Patient Movement (Moving from one clinic to another) - Socio-economic issues (access to healthcare; long distance to hospital)
https://doi.org/10.1177/23259582211052399	Manavalan et al. [61]	- Patient-level barriers: Stress, depression, and HIV-related stigma; lack of knowledge and awareness about hypertension - Provider-level barriers: Inadequate hypertension training and knowledge gaps; ineffective antihypertensive prescribing practices; challenges with hypertension counseling - System-level barriers: Limitations in clinic capacity for hypertension care; high costs of hypertension care; lack of routine hypertension screening and monitoring; challenges with hypertension data, guidelines, and protocols

## Discussion

To our knowledge, this mixed systematic review with meta-analysis is the second of its kind conducted in Sub-Saharan Africa [15]. However, it uniquely addresses the challenges faced in the hypertension prevention cascade among people living with HIV (PLWH).

### Prevalence of Hypertension Among PLWH in Sub-Saharan Africa

The results of this meta-analysis reveal a hypertension prevalence of 25% (95% CI: 21%–28%) among 32,800 people living with HIV (PLHIV) attending routine HIV care in clinics across 15 sub-Saharan African (SSA) countries. While this prevalence aligns with previously documented trends, our study updates and strengthens the evidence base using more recent data. Four prior systematic reviews have assessed hypertension prevalence in PLHIV: a 2015 review first reported rates ranging from 8.7% to 45.9% in middle-income countries [65]. A 2017 meta-analysis of 49 studies, including 10 from Africa, estimated an overall prevalence of 25.2% (95% CI: 21.2%–29.6%) [4]. More recently, Ataklte et al. (2021) analyzed 194 studies comprising 396,776 PLHIV from 61 countries and found a global prevalence of 23.6% (95% CI: 21.6%–25.5%), with 23.5% (95% CI: 16.6%–31.0%) in West and Central Africa [66]. Additionally, a 2023 systematic review focusing on 23 studies in SSA reported a regional prevalence of 19.6% (95% CI: 16.6%–22.5%) [67]. These findings confirm that SSA is facing a dual epidemiological burden, wherein hypertension affects nearly one in four PLHIV. This reinforces the urgency of integrating non-communicable diseases (NCDs) into HIV care, as recommended by the WHO, which calls for routine screening for hypertension at every clinic visit for adult PLHIV [68].

### Treatment and Control of Hypertension

Beyond estimating prevalence, our findings highlight a critically deficient hypertension care cascade among PLHIV in SSA. While 95% (95% CI: 89%–100%) of individuals were screened, only 20% (95% CI: 8%–33%) of those diagnosed received treatment, and just 19% (95% CI: 3%–34%) achieved blood pressure control. This stark disconnect has been echoed in recent cohort studies [69, 70] and reflects a structural gap between detection and clinical response. Commonly reported barriers include the limited availability of antihypertensive medications [71], the lack of integrated treatment algorithms within HIV services [72], insufficient healthcare worker training on NCD management [73], and the absence of dedicated funding for integrated care models [74]. The failure to translate screening into actionable clinical interventions has already been described as a major threat to the cardiovascular health of PLHIV [75].

Furthermore, the high degree of statistical heterogeneity (I^2^ > 97%) across all cascade steps reflects significant contextual variation between countries, health systems, available resources, and patient characteristics. This supports conclusions [76], which documented significant disparities in the integration of HIV and NCD care, with more advanced implementation in urban areas, middle-income countries, or facilities supported by international donors. Notably, differences in national health policies may contribute to these variations. For instance, countries like South Africa have made significant strides in integrating chronic disease management into routine HIV services, including the adoption of national guidelines recommending systematic hypertension screening and management in HIV care settings. Such policy frameworks may partly explain the relatively better hypertension outcomes observed in these countries compared to others with less structured integration [77]. Our subgroup analyses also revealed that age was significantly associated with treatment initiation (p = 0.04), consistent with clinical recommendations that prioritize cardiovascular prevention in older adults [56]. Although gender did not significantly influence treatment initiation, it was associated with differences in blood pressure control (p = 0.02). These gender-related disparities may be attributed to differences in adherence, pharmacologic responsiveness, or systemic biases in care delivery, as previously documented in several meta-analyses [78, 79].

In summary, our study highlights the systemic shortcomings in the integration of HIV and NCD care across SSA. While screening coverage is relatively high, it remains ineffective without actionable responses at the treatment and follow-up levels. Innovative and targeted strategies are urgently needed, such as deploying community health workers for blood pressure monitoring [80], incorporating antihypertensive drugs into antiretroviral supply chains [81], and digitizing patient records to strengthen longitudinal follow-up [82]. The functional integration of HIV and NCD services must evolve from a programmatic ambition to a political and operational priority. As Doshi et al. (2023) underscore, strengthening this integration could not only improve cardiovascular outcomes for PLHIV but also enhance health system resilience in the face of future health crises [83].

### Challenges in Hypertension Prevention

This review is the first of its kind to compile scientific evidence addressing the challenges of hypertension prevention among PLWH in Sub-Saharan Africa. We identified 08 qualitative and quantitative studies addressing this issue, which revealed various types of challenges. We categorized these obstacles into three groups: patient-related challenges, provider-related barriers, and systemic healthcare challenges.

#### Patient-Level Challenges

Stress, depression, HIV-related stigma, lack of knowledge about hypertension, self-stigmatization, and poor adherence to antihypertensive medication were identified as patient-related challenges. Stigma and mental health issues, such as depression, were recognized as significant barriers to hypertension care at the patient level [84]. Synthesized studies suggested that HIV-related stigma leads to increased stress and depression, potentially resulting in disengagement from the medical system and subsequent non-adherence to antihypertensive treatment. It is well established that HIV stigma negatively impacts engagement in HIV care and adherence to antiretroviral therapy [85]. Moreover, evidence suggests that HIV stigma is linked with disengagement and poor clinical outcomes for other chronic diseases [86]. Given the rising prevalence of cardiovascular diseases among individuals with infectious diseases, these findings emphasize the necessity of addressing stigma and mental health to enhance engagement in NCD care. Several studies reported that patients lacked knowledge regarding hygiene and dietary measures. Low awareness of risk factors, signs, and management of hypertension was observed, consistent with other studies in the region [87–89]. Insufficient and inaccurate knowledge about hypertension among patients was identified as a primary barrier to awareness, treatment, and control in multiple studies. Universally, all studies noted a lack of knowledge among PLWH [59]. Conversely, studies highlighted a good understanding of HIV infection and treatment among patients, likely due to concentrated efforts in HIV education and large-scale awareness campaigns in Sub-Saharan Africa. This underscores the importance of health education initiatives in treatment adherence and clinical outcomes. Several studies in the region have demonstrated improved hypertension outcomes following educational interventions [89, 90]. Therefore, implementing patient-directed educational interventions on hypertension, both in community settings and clinical contexts, may enhance cardiovascular outcomes among PLWH. Given the success of peer education in HIV management in Sub-Saharan Africa [91, 92], the role of peer educators in managing NCDs warrants serious consideration and further research.

#### Provider-Level Barriers

Provider-related gaps were identified as challenges limiting hypertension prevention among PLWH, including insufficient counseling on hypertension, lack of knowledge regarding management and prevention, high consultation costs with specialists, and the absence of standardized hypertension diagnostic algorithms or protocols within HIV prevention clinics. In Tanzania, a study by *Manavalan and al* in 2021 highlighted several provider-level challenges, including personnel shortages, high costs of hypertension care, inadequately equipped facilities for hypertension management, lack of systematic hypertension screening, and insufficient funding for NCDs [57]. These findings align with data exploring barriers to hypertension care faced by the general population in Sub-Saharan Africa [93, 94]. Some studies also indicated that systemic challenges are the underlying causes of many patient- and provider-level barriers. In Cameroon, Tanzania, and South Africa, clinician shortages combined with high patient volumes have restricted screening, management, and follow-up practices for hypertension. These gaps subsequently impact patient knowledge of hypertension and adherence to treatment. Moreover, many of the described barriers were specific to hypertension and not encountered in HIV care; for instance, the high cost of hypertension care is a distinct barrier for NCDs, as all HIV-related services in African countries are provided free of charge [87]. HIV prevention programs in Sub-Saharan Africa have been successful and are universally subsidized. These investments positively impact clinical outcomes throughout the HIV treatment cascade. For instance, in Cameroon in 2023, 95% of individuals knew their HIV status, new infections were reduced by 31.6%, mortality declined by 23.9%, and the quality of life of PLWH significantly improved due to various implemented strategies [95]. In contrast, a study conducted in Cameroon involving over 8,000 hypertensive HIV patients reported catastrophic outcomes concerning screening, treatment, and control [11]. To eliminate the barriers encountered throughout the hypertension treatment cascade, prioritization and funding for integrating chronic disease prevention into HIV control programs are essential. Integrating NCD care into existing HIV clinical programs has proven to be a promising approach to improve hypertension outcomes among PLWH, leveraging existing infrastructure and avoiding additional burdens on an already strained system [39]. Furthermore, utilizing the strengths of HIV programs in managing non-communicable diseases aligns with WHO strategic guidelines.

#### System-Level Barriers

In this category, challenges identified in the studies were classified into six groups, including the lack of a continuum of integrated hypertension prevention care in HIV clinics, unavailability and high costs of medications, absence of patient education programs, distances to health facilities, high transport costs, lack of a well-defined national policy on non-communicable disease prevention, and low patient awareness. In Tanzania, healthcare providers reported in 2021 that system-level obstacles were the most significant barriers to hypertension care among PLWH. Among systemic challenges, they listed personnel shortages, high costs of hypertension care, inadequately equipped facilities for hypertension management, lack of systematic hypertension screening, and insufficient funding for non-communicable diseases [59]. In Cameroon, there is currently no national hypertension prevention program available. Nevertheless, a pilot project is being implemented in Cameroon and Senegal to prevent and manage non-communicable diseases among PLWH, monitored at the Military Hospital of Yaoundé and the Regional Annex Hospital of Bafia. Preliminary results from this project indicate the urgent need for the government to draft national policies for hypertension and NCD prevention and that integrating hypertension and diabetes care into HIV clinics is effective [96, 97].

### Strengths and Limitations

The strength of this study lies in its integration of both quantitative and qualitative evidence addressing a range of topics, including the epidemiological distribution of hypertension (HTN) among people living with HIV (PLWH), the cascade of prevention data, and the challenges encountered in preventing HTN among PLWH in Sub-Saharan Africa (SSA). This represents a significant first in this context. However, this systematic review does have certain limitations. Firstly, the absence of studies from some SSA countries may lead to a partial and unrepresentative view of the overall situation. Additionally, all included studies focused on a limited sample of PLWH in SSA. A second limitation pertains to potential biases in the meta-analysis of HTN screening data among PLWH, from which one study was excluded. The scarcity of data on the treatment and control of HTN also represents a limitation that could affect the findings reported in this review, particularly if new data becomes available.

### Conclusion

This mixed-methods systematic review with meta-analysis highlights the high prevalence of HTN and the substantial gaps in the treatment and control of HTN among PLHIV in sub-Saharan Africa. Our findings reveal significant heterogeneity between countries, driven by contextual differences in national policies, health infrastructure, individual factors, and the degree of integration between HIV and NCD care. The qualitative synthesis identified both systemic and behavioral barriers, including low patient awareness, limited access to integrated services, insufficient human resources, and reduced motivation for preventive behaviors among both providers and patients. To overcome these challenges, the systematic integration of HTN screening and management into HIV care services leveraging existing infrastructure is essential. This review supports the implementation of evidence-based interventions tailored to the African context [98]. The HTN prevention measures proposed by the African Society of Hypertension [99] provide a relevant operational framework for integrating HTN services into HIV programs. Furthermore, the VIHEILLIR project in Cameroon [96], which promotes healthy aging with HIV through cardiovascular risk reduction, and the WHO’s HEARTS strategy currently being piloted at the Yaoundé Central Hospital illustrate scalable, context-sensitive approaches. Scaling up such interventions, alongside community-based non-pharmacological strategies developed by African researchers, will be crucial to reversing current trends and improving cardiovascular outcomes among PLHIV in the region.
