# Supplementary material for: Burden and Challenges of Managing Hypertension in People Living with Human Immunodeficiency Virus (HIV) Infection in Sub-Saharan Africa: A Mixed Systematic Review and Meta-Analysis
Source: Int J Public Health. 2025 Dec 17;70:1608521. doi: 10.3389/ijph.2025.1608521 (PMC12753512; doi:10.3389/ijph.2025.1608521)
Supplement: Supplementary file 1 [file DataSheet1.pdf]

Burden and Challenges of Managing Hypertension in People Living with HIV in Sub-Saharan Africa: A Mixed Systematic Review with Meta-Analysis

Francis Duhamel NANG NANG[1,2,3], Anastase DZUDIE[4,5,6,7], Liliane KUATE MFEUKEU[7,8], Paul Junior CHEBO[1] Rita Marie IFOUE[1], Jean Pierre Junior TCHITETCHOUN[1], François Anicet ONANA AKOA[9,10], André Pascal KENGNE[4,6,11], Charles KOUANFACK[1,12,13], Simeon Pierre CHOUKEM[1,14,15].

Table of contents

Supplementary Figures S1 : Age subgroup plot of Percentage of PLHIV with confirmed HTN 2
Supplementary Figures S2 : Gender subgroup plot of Percentage of PLHIV with confirmed HTN 2
Supplementary Figures S3 : Age subgroup plot of Screening proportion 3
Supplementary Figures S4 : Gender subgroup plot of Screening proportion 3
Supplementary Figures S5 : Age subgroup plot of Treatment initiation proportion.................... 4
Supplementary Figures S6 : Gender subgroup plot of Treatment initiation proportion 4
Supplementary Figures S7 : Gender subgroup plot of Hypertension control proportion 5
Supplementary Figures S8 : Age subgroup plot of Hypertension control proportion 5

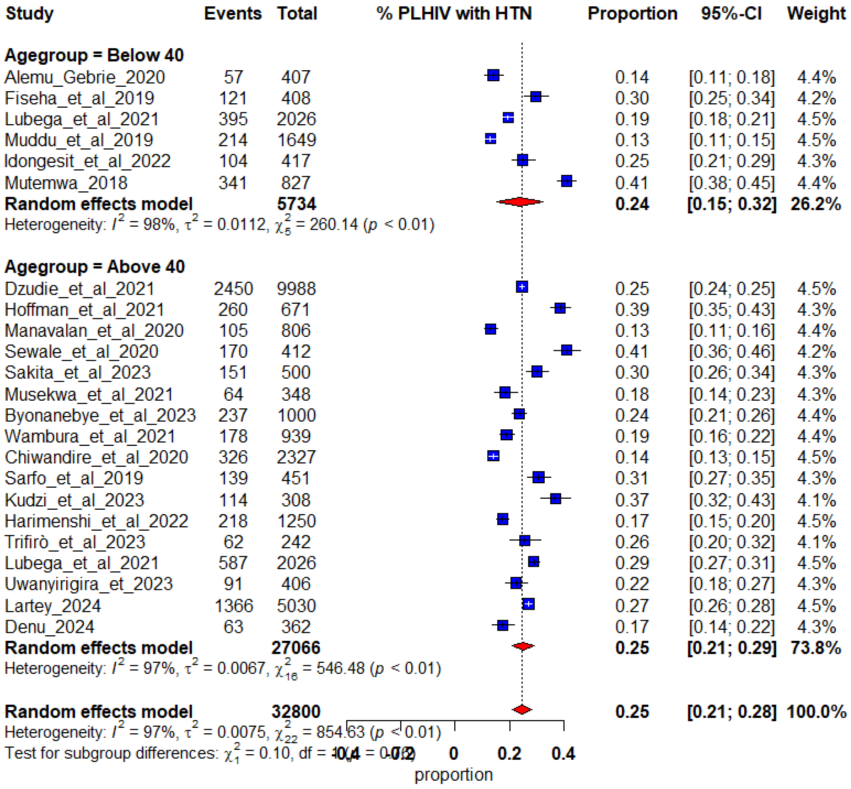

Supplementary Figures S1 : Age subgroup plot of Percentage of PLHIV with confirmed HTN

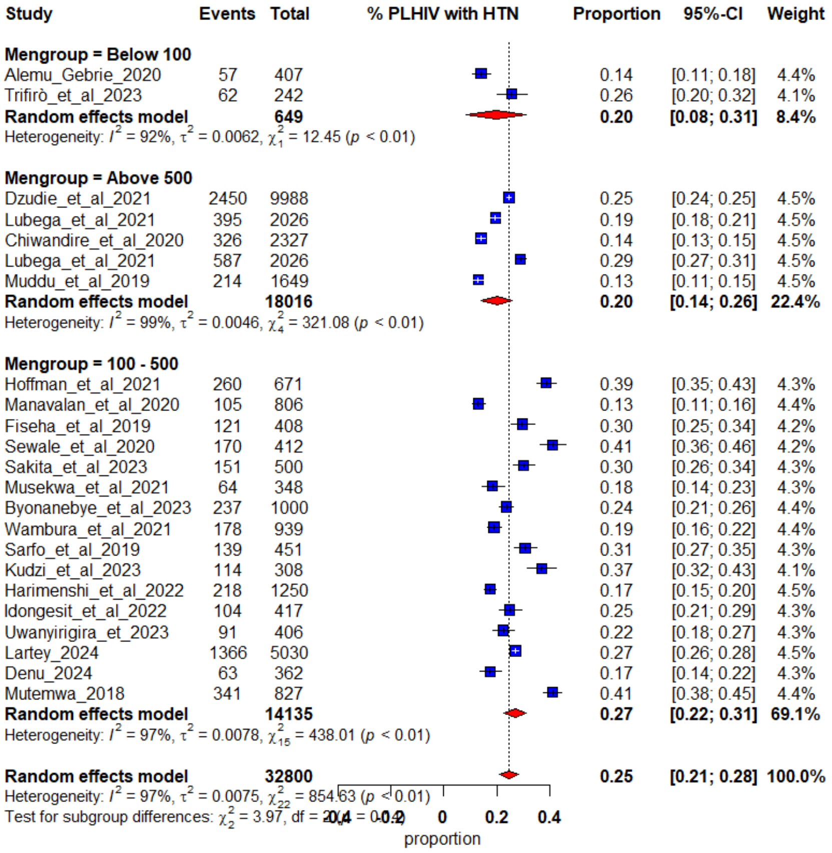

Supplementary Figures S2 : Gender subgroup plot of Percentage of PLHIV with confirmed HTN

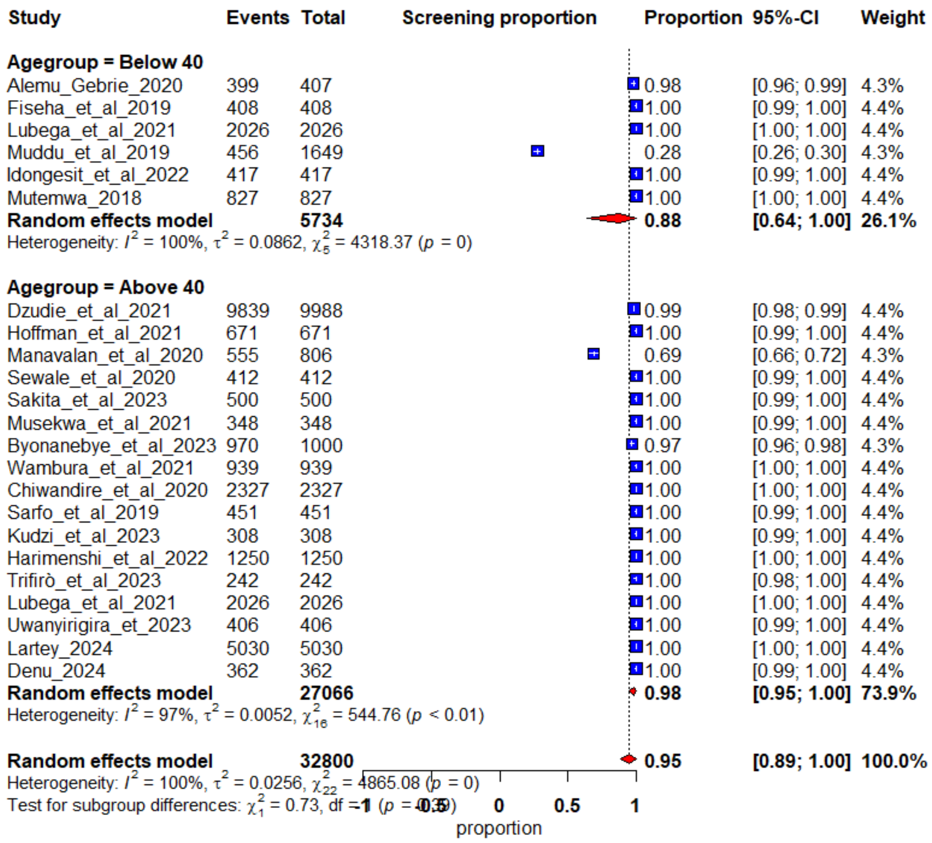

Supplementary Figures 3 : Age subgroup plot of Screening proportion

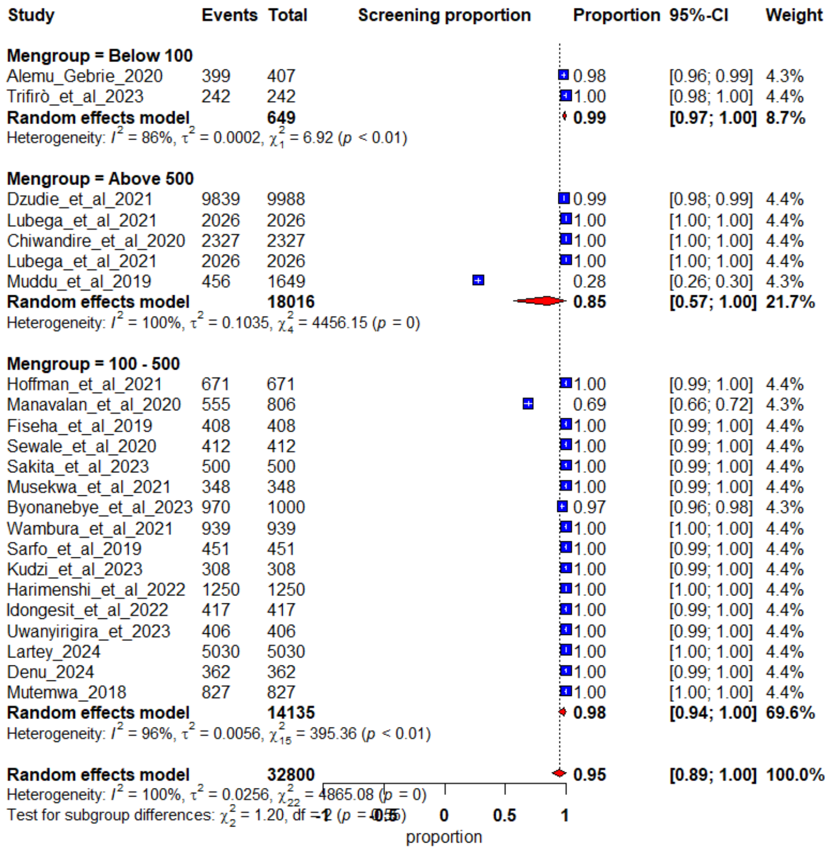

Supplementary Figures S4 : Gender subgroup plot of Screening proportion

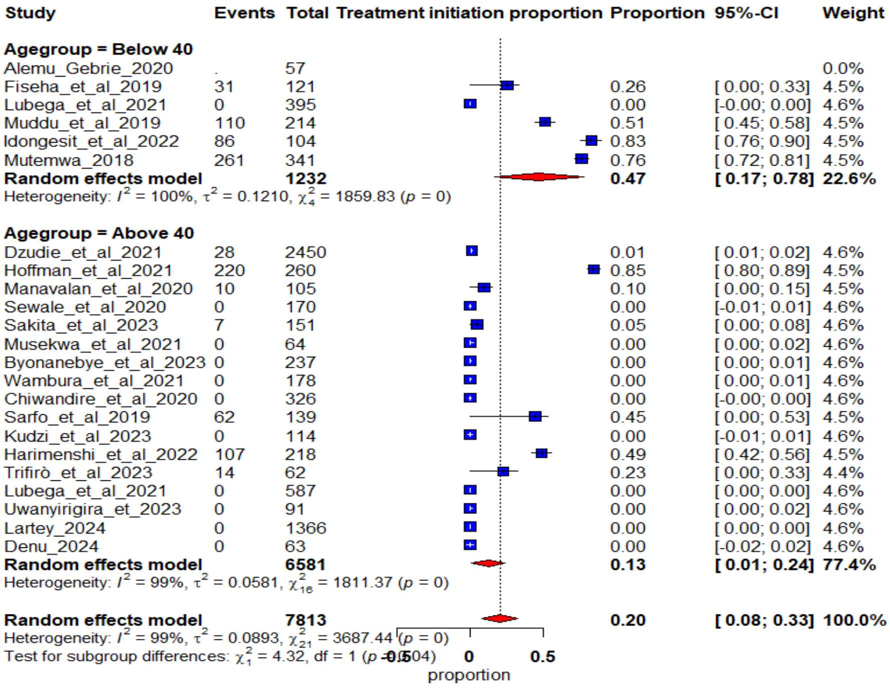

Supplementary Figures S5 : Age subgroup plot of Treatment initiation proportion

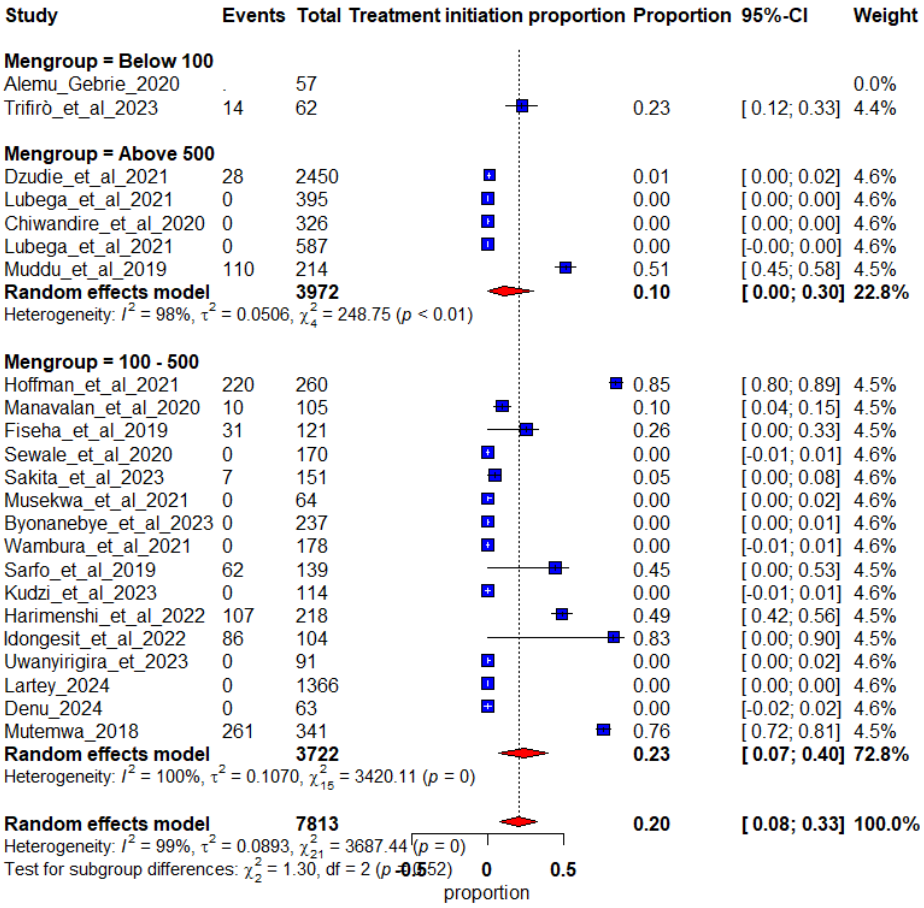

Supplementary Figures S6 : Gender subgroup plot of Treatment initiation proportion

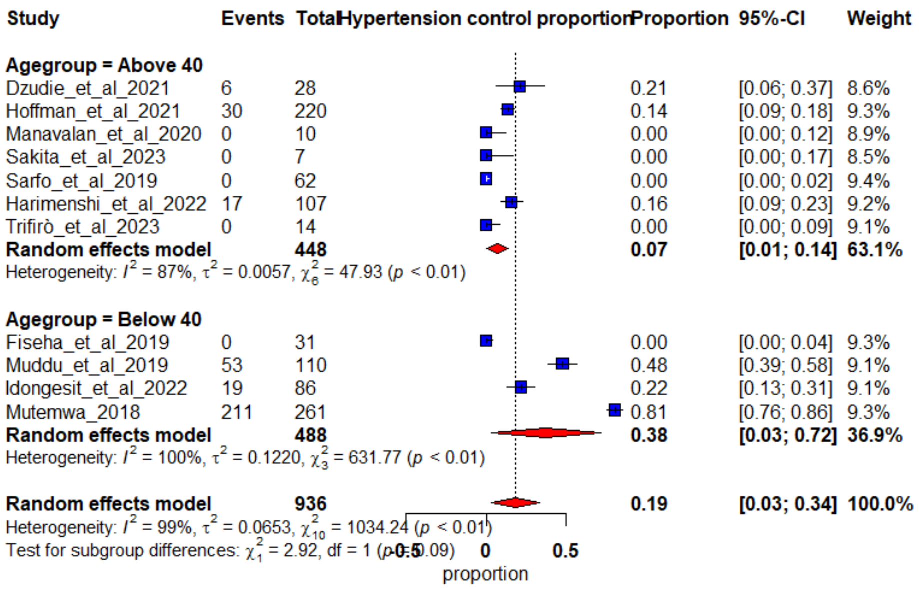

Supplementary Figures S7 : Age subgroup plot of Hypertension control proportion

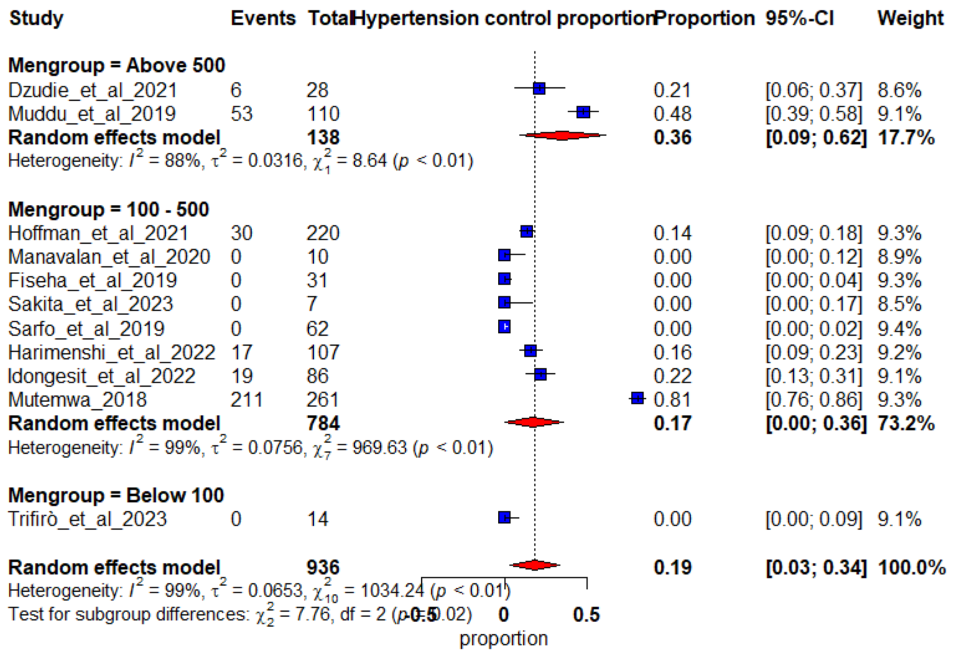

Supplementary Figures S9: Gender subgroup plot of Hypertension control proportion
